# A Non-Neuronal Cardiac Cholinergic System Plays a Protective Role in Myocardium Salvage during Ischemic Insults

**DOI:** 10.1371/journal.pone.0050761

**Published:** 2012-11-29

**Authors:** Yoshihiko Kakinuma, Tsuyoshi Akiyama, Kayo Okazaki, Mikihiko Arikawa, Tatsuya Noguchi, Takayuki Sato

**Affiliations:** 1 Department of Cardiovascular Control, Kochi Medical School, Nankoku, Kochi, Japan; 2 Department of Cardiac Physiology, National Cardiovascular Center Research Institute, Suita, Osaka, Japan; Thomas Jefferson University, United States of America

## Abstract

**Background:**

In our previous study, we established the novel concept of a non-neuronal cardiac cholinergic system–cardiomyocytes produce ACh in an autocrine and/or paracrine manner. Subsequently, we determined the biological significance of this system–it played a critical role in modulating mitochondrial oxygen consumption. However, its detailed mechanisms and clinical implications have not been fully investigated.

**Aim:**

We investigated if this non-neuronal cardiac cholinergic system was upregulated by a modality other than drugs and if the activation of the system contributes to favorable outcomes.

**Results:**

Choline acetyltransferase knockout (ChAT KO) cells with the lowest cellular ACh levels consumed more oxygen and had increased MTT activity and lower cellular ATP levels compared with the control cells. Cardiac ChAT KO cells with diminished connexin 43 expression formed poor cell–cell communication, evidenced by the blunted dye transfer. Similarly, the ChAT inhibitor hemicholinium-3 decreased ATP levels and increased MTT activity in cardiomyocytes. In the presence of a hypoxia mimetic, ChAT KO viability was reduced. Norepinephrine dose-dependently caused cardiac ChAT KO cell death associated with increased ROS production. In *in vivo* studies, protein expression of ChAT and the choline transporter CHT1 in the hindlimb were enhanced after ischemia-reperfusion compared with the contralateral non-treated limb. This local effect also remotely influenced the heart to upregulate ChAT and CHT1 expression as well as ACh and ATP levels in the heart compared with the baseline levels, and more intact cardiomyocytes were spared by this remote effect as evidenced by reduced infarction size. In contrast, the upregulated parameters were abrogated by hemicholinium-3.

**Conclusion:**

The non-neuronal cholinergic system plays a protective role in both myocardial cells and the entire heart by conserving ATP levels and inhibiting oxygen consumption. Activation of this non-neuronal cardiac cholinergic system by a physiotherapeutic modality may underlie cardioprotection through the remote effect of hindlimb ischemia-reperfusion.

## Introduction

Our previous studies using animal models of heart failure [1]–[6] and angiogenesis [7] focused on whether manipulating the parasympathetic nervous system may offer a beneficial therapeutic modality against cardiovascular diseases. Subsequently, we found that vagal nerve stimulation [4], [6] and the acetylcholinesterase inhibitor donepezil [8], [9] activated an ischemia or hypoxia-resistant system independent of their heart rate reduction effects [1], [4], [10] and provided a promising outcome by slowing the progression of cardiac remodeling associated with chronic heart failure. We also found that donepezil played a role in accelerating angiogenesis in a murine hindlimb ischemia model by stimulating angiogenic mechanisms and alternatively by inhibiting ischemic skeletal muscle cell apoptosis [7].

However, despite the convincing data for vagal nerve stimulation in chronic heart failure, an anatomical characteristic of vagal nerve innervations of the heart, i.e., an extremely low density of vagal nerve ends in the cardiac ventricles, prompted us to consider that there was a missing link. Consequently, our recent study presented a novel concept that cardiomyocytes synthesize ACh themselves and that the synthesis is enhanced by ACh or a muscarinic receptor agonist [11]. Based on the results of this study, a non-neuronal cardiac cholinergic system exists in cardiomyocytes and produces measurable levels of ACh. In addition, cardiomyocyte-derived ACh downregulates mitochondrial function to circumvent mitochondrial overshoot [11]. This study clearly indicated that the non-neuronal cardiac cholinergic system protected cardiomyocytes from energy depletion when the cardiac energy demand was enhanced, e.g., increased oxygen consumption due to a pathologically elevated cardiac workload. Therefore, this system would be expected to be a barrier against hypoxic or reactive oxygen species stress because locally synthesized ACh would sequentially activate and amplify this system in an autocrine and paracrine manner into the entire heart [11].

However, the following issues remain to be clarified. First, how is this system essentially involved in vital functions of cells, specifically, whether cells can perform ordinary biological functions without ACh? Second, how is the system important in pathological condition? Third, how is this system actively modulated by non-medical means and whether its upregulation can protect the heart *in vivo* from pathological insults?

In this study, to clarify these issues, we performed experiments using siRNA to generate ChAT knockout (ChAT KO) cells. The phenotypes of ChAT KO cells derived from epithelium or myocardium were compared with those from wild-type cells. We also investigated whether a non-medical means, i.e., preconditioning through hindlimb ischemia-reperfusion (IR), upregulated a non-neuronal cholinergic system in the heart, which was a remote organ from the hindlimb skeletal muscles. Finally, we examined whether such a remote activation of this system in the heart may provide cardioprotection from myocardial ischemia.

## Methods

### 1. Animals

All animal procedures using Male C57BL/6 mice (Japan SLC inc., Hamamatsu, Japan) aged between 9 and 11 weeks (20–25 g) were performed in strict accordance with the recommendations in the guidelines of the Physiological Society of Japan and the protocols were approved by the Animal Research Committee of Kochi Medical School (Permit Number: E-00017). All surgery was performed under sodium pentobarbital anesthesia, and all efforts were made to minimize suffering.

### 2. ChAT Gene Knockout

ChAT gene knockout was performed using a BLOCK-iT™ Pol II miR RNAi expression vector, pcDNA™ 6.2-GW/EmGFP-miR (Invitrogen Corporation, Carlsbad, CA, USA). According to the manufacturer’s protocol, commercially recommended and pre-designed RNAi sequences specific for human or mouse ChAT were used to prepare double-stranded oligos, which were subcloned into the expression vector. As a negative control, Lac Z specific RNAi sequences were subcloned to develop a negative control expression vector, which was also commercially recommended. To confirm that ChAT gene expression was suppressed, HEK293 cells that were transiently transfected using Effectene transfection reagent (QIAGEN), were examined by immunocytochemical studies. Stable transfectants of ChAT KO expression vectors were developed with appropriate selective antibiotics in HEK293cells (ChAT KO HEK cells) and in HL-1 cells (ChAT KO HL-1 cells), which were derived from murine atrial myocardium. Transfected cells were easily detected because of their expression of GFP.

### 3. Immunocytochemical Studies

The following primary antibodies were used in immunocytochemical analysis: a goat anti-ChAT polyclonal antibody (Millipore, Billerica, MA, USA) at 1∶200 dilution; a rabbit anti-catenin polyclonal antibody (Cell Signaling Technology, Inc., Danvers, MA, USA) at 1∶200 dilution; rat anti-ACh polyclonal antibody (Millipore) at 1∶500 dilution; and rabbit anti-Cx43 polyclonal antibody (Zymed Laboratories Inc., South San Francisco, CA, USA). After overnight incubation at 4°C, washing with PBS, an appropriate secondary antibody conjugated with an immunofluorescent tag was applied. Samples were examined by laser confocal microscopy (Olympus, Tokyo, Japan).

Intensity or linear length of the immunoreactivity was semi-quantitatively measured using an image-analysis system (Image J, National Institute of Health, USA). For proper comparison, multiple cells (300–400 cells in total) in at least 3 randomly selected visual fields were evaluated under the comparable conditions for the confocal microscopic examination between control cells and ChAT KO cells. In addition, cells expressing strong β-catenin signals with apparently linear forms were counted and the rate in each visual field was calculated.

### 4. Western Blot Analysis

Western blot analysis was performed as described in our previous studies [2], [3], [7], [10], [11]. The following primary antibodies were used: a goat anti-ChAT polyclonal antibody (Millipore) at 1∶500 dilution; a rabbit anti-CHT1 polyclonal antibody (Antagene, Inc., Limonest, France) at 1∶500 dilution; and a rabbit anti-cleaved caspase-3 monoclonal antibody (Cell Signaling Technology, Inc.) at 1∶500 dilution. After reaction with an appropriate HRP-conjugated secondary antibody followed by washing with 1% TBST, signals were detected using Luminata™ Forte Western HRP Substrate (Millipore). Representative data were shown from independently performed experiments (n = 4–6).

### 5. LY Dye Transfer Assay

To assess the effects of the cardiac non-neuronal cholinergic system on cardiomyocyte-derived cells, we generated permanent ChAT KO HL-1 cells. These cells were placed onto glass bottom dishes, such that the cells were aligned linearly at 60 µm in width (Cyto Graph, Dai Nippon Printing Co., Ltd., Tokyo, Japan). Each band of linearized ChAT KO HL-1 cells was patterned in parallel approximately 200 µm apart from each other. The linearized cells were scratched perpendicularly using a 27-gauge needle after removing culture medium, and Lucifer yellow dye (1%) was applied to the center of the scratched area. This method was already known as a scrape and scratch method for evaluating a gap junction function [3], [12]. The dye does not diffuse through intact plasma membrane, but its low molecular weight permits the transmission across patent gap junctions, once it is introduced to injured cardiomyocytes. After 1 minute, the cells were washed thrice with PBS, fixed with 4% paraformaldehyde for 10 min, after which the distance that the dye had spread from the scratched region was evaluated using an immunofluorescent microscope.

### 6. Cell Culture

To investigate the fundamental significance of a non-neuronal cholinergic system, which may be generally equipped by other cells [13], we used several types of cells, not only cardiomyocyte-derived cells but also other cells with the fact that specific cells (HEK293 cells, purchased from DS Pharma Biomedical Co., Ltd., Suita. Osaka, Japan) are very useful for gene transfer experiments in mind. HEK293 cells and H9c2 cells, which were spontaneously immortalized ventricular myoblasts from rat embryos, purchased from DS Pharma Biomedical, were cultured in DMEM (Wako Pure Chemical Industries, Ltd., Osaka, Japan) supplemented with 10% FBS and antibiotics. HEK293 cells were cultured on culture dishes coated with type I collagen (Cellmatrix, Nitta Gelatin Inc., Osaka, Japan). HL-1 cells, derived from atrial cardiac muscle, gift from Dr. William Claycomb (Department of Biochemistry & Molecular Biology, Louisiana State University Medical Center, New Orleans) were cultured in Claycomb medium (SAFC Biosciences, Inc., Lenexa, KS, USA) supplemented with 10% FBS, 4 mM L-glutamine, 0.1 mM norepinephrine and 3 M L-ascorbic acid on the culture dishes coated with 0.02% gelatin and 25 g/mL of fibronectin [14]. To inhibit cellular ChAT activity, cultured cells were treated with 10 µM of hemicholinium (HC)-3 during experiments, the dose of which was confirmed to be not harmful to cells.

### 7. ACh Concentrations in Cell Lysates

As reported in our recent study, the cellular or whole-heart ACh concentration was determined by HPLC [11]. To prepare cell lysates, cells were scraped from a 10-cm culture dish, washed by PBS, and then suspended in 1 mL of PBS containing 0.1 mM physostigmine and 2×10^−8^ M IPHC as an internal control. These samples were then freeze-thawed thrice. A heart excised from a humanely sacrificed mouse was thoroughly minced and homogenized in the presence of 1 mL of homogenate solution that included 0.1 M perchloric acid, 0.1 mM physostigmine, and 2×10^−8^ M IPHC. After centrifugation at 0°C at 2×10^4^ g for 15 min, the pH of the supernatant was adjusted with 1 M potassium bicarbonate and then filtered using an Ultrafree MC column (Millipore). The eluted solution was used to quantitatively determine the ACh concentration.

### 8. MTT Activity

MTT activity was determined using Cell Counting Kit-8 (Dojindo Laboratories, Kumamoto, Japan) according to the manufacturer's protocol. MTT activity is known to be dependent on cell viability (the number of intact cells). However, it was reported that MTT activity was determined by the cell number as well as by cell metabolism [10], [15]. Specifically, MTT activities of cells with malfunctioning mitochondria showed a trend for reduced activity even with comparable cell numbers.

### 9. Cellular Oxygen Consumption

Oxygen concentrations in culture medium were determined by Fibox 3 (PreSense Precision Sensing, Regensburg, Germany), as in our previous study [11]. A specific oxygen-sensitive sensor was attached at the bottom of a culture dish, after which cells were incubated in this dish. Oxygen concentration was determined sequentially, recorded and compared with that at 0 h.

### 10. Cellular ATP Concentration

Cellular and heart ATP levels were determined using an XL-ATP kit (APRO Life Science Institute, Tokushima, Japan) according to the manufacturer’s protocol. The ATP level of cell lysates was corrected by the protein concentration.

### 11. Cellular Reactive Oxygen Species Production

Using an indicator for reactive oxygen species (ROS), aminophenyl fluorescein (APF, Sekisui Medical Co., Ltd., Tokyo, Japan), we evaluated ROS production by ChAT KO HL-1 cells or control HL-1 cells in the presence of different concentrations of norepinephrine (0.5, 1.0, and 2.0 mM). APF was added to culture medium at a final concentration of 10 µM. Cells were incubated with APF for 1 h and observed by laser confocal microscopy.

### 12. Global Ischemia Model of the Murine Heart with a Langendorff Apparatus

After anesthesia with an intraperitoneal injection of pentobarbital, a male C57BL/6J mouse was subjected to left hindlimb ischemia (5 min)-reperfusion (3 min) (IR), which was repeated thrice and performed by a physical rather than a surgical procedure. A ChAT inhibitor hemicholinium (HC) -3 was intraperitoneally administered soon after IR. The right and left quadriceps femoris muscles were excised to assess ChAT and CHT1 protein expression 16 h after reperfusion. This time point for sampling was determined on the basis of the time course experiment, where after reperfusion for 4 h, 8 h, or 16 h, a heart or skeletal muscle was excised to evaluate protein expression. As shown, those expression levels were identified to peak at 12 h, therefore, we chose to sample tissue after 16 h.

To exclude the possibility that ACh could be derived from a neuronal origin, i.e., vagal nerve endings in the heart, a Langendorff apparatus was useful for such an experiment, because the heart was completely isolated from the regulation of the cholinergic system. Moreover, due to the murine heart-specific innervation mode, i.e., the very poor vagal nerve innervation in the heart and the excised heart perfused for 1.5 h without nerve regulation, such experimental conditions might not allow us to consider that secreted ACh from neurons could protect the heart. Another heart excised 16 h after the ischemia-reperfusion was connected to a Langendorff apparatus and perfused with filtered Krebs–Henseleit buffer at 90 mmHg, equilibrated with 5% carbon dioxide and 95% oxygen. After stabilization, the Langendorff-perfused heart was subjected to 30 min of global ischemia by stopping the Krebs–Henseleit buffer perfusion, followed by 60 min of reperfusion. Subsequently, the heart was immersed in a 1% TTC staining solution for 10 min at 37°C, after which the stained heart was horizontally sliced in the mid-body and the infarcted area was evaluated. The proportion of the infarcted area was determined by dividing the infarcted area by the whole area using NIH image software.

### 13. Statistical Analysis

Data were expressed as mean ± standard error. A non-parametric Mann-Whitney U-test was used to compare two groups. Non-parametric multiple-comparison tests among three groups used Kruskal-Wallis test followed by Fisher’s PLSD tests. Differences were considered significant at *P*<0.05.

## Results

### 1. The Non-neuronal Cholinergic System Regulates Cell–cell Communication

**Figure 1:**
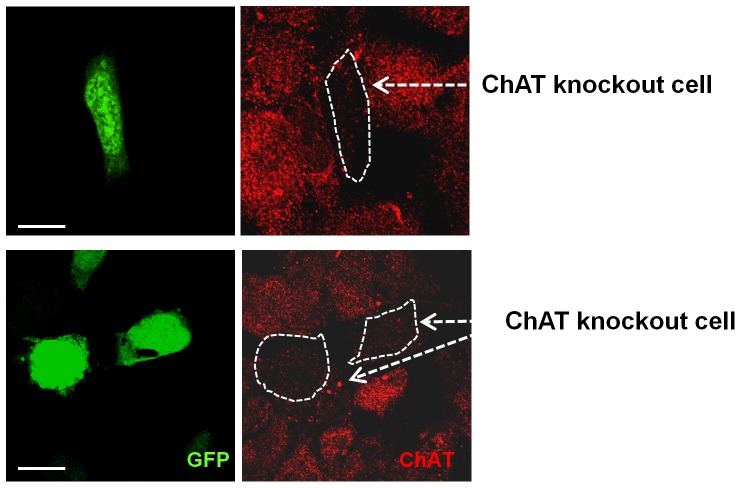
Validation of ChAT KO using HEK 293 cells, which were transiently transfected by a ChAT−specific miR RNAi expression vector. ChAT KO cells, transiently transfected with a ChAT−specific miR RNAi expression vector, surrounded by white dotted lines, also detected by the GFP signals (green), showed attenuation of ChAT immunoreactivities (red dots hardly detected in a ChAT KO cell accompanying a white arrow). In contrast, native ChAT signals in non-transfected cells were detected with red dots (native ChAT signals). Scale bar: 10 µm. Representative data were shown from 4 independently performed experiments.

To determine the specific roles of the non-neuronal cholinergic system *in vitro*, the ChAT gene was knocked out with a ChAT-specific miRNA expression vector. Permanent ChAT KO cells were prepared using HEK293 cells and HL-1 cells, which were derived from murine atrial myocardium. To verify the effects of ChAT knockdown vectors currently used, ChAT gene knockout was immunocytochemically confirmed in transiently transfected HEK293 cells by their GFP expression (Figure 1; green staining). ChAT immunoreactivity, as observed in non-transfectants (red), was completely attenuated in ChAT KO cells, suggesting that this knockdown vector worked to suppress ChAT gene expression.

**Figure 2:**
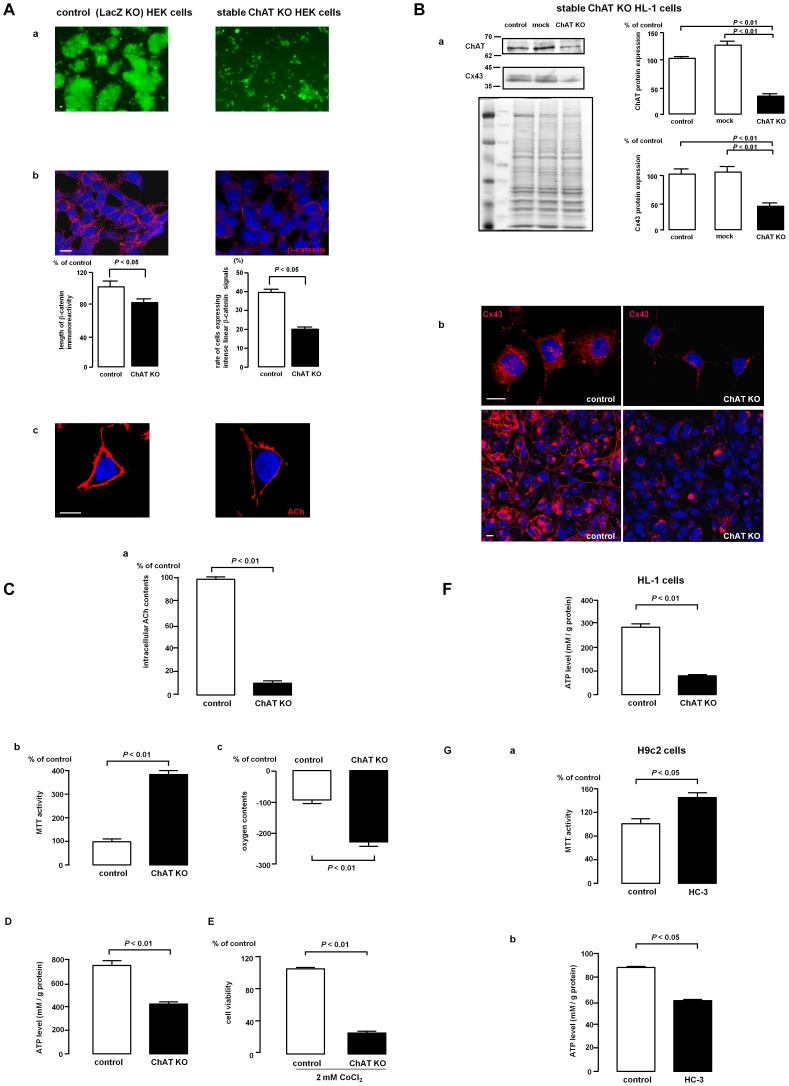
ChAT gene deletion affects cell−cell communication through β-catenin and Cx43, and modulates cellular energy metabolism. (A) Stably transfected ChAT KO cells with reduced β-catenin immunoreactivity (signal intensity: 80.7±4.7% vs. control, *P*<0.05; rate with the linear form: 20.1±2.2% vs. control, *P*<0.05) observed between cells (A−b) were easily separated by simple agitation without forming large aggregates as compared with control LacZ KO cells (A−a). The intensities and lengths of β-catenin signals with linear forms were measured using Image J, and the rate of cells expressing strong β-catenin signals in the intercellular spaces were calculated (A−b). ChAT KO cells exhibited fewer ACh immunoreactive signals at the cell membrane (A−c). Representative data from 5 independent experiments were shown. Scale bar: 10 µm. (B) ChAT KO HL-1 cells (murine cardiomyocyte-derived HL-1 cells stably transfected with ChAT specific miR RNAi expression vector) expressed less Cx43 compared with control LacZ KO HL-1 cells, as shown by western blot (Cx43: 48.7±5.0% vs. control, *P*<0.01) (B−a) and immunocytochemistry (B−b). The Cx43 signals in cytoplasm and intercellular spaces were attenuated in ChAT KO HL-1 cells compared with control cells (signal intensity: 41.6±5.1%, *P*<0.05; signal length: 25.8±1.5%, *P*<0.05) (B−b). The signal intensities and lengths of Cx43 signals were evaluated by Image J. Representative data from 5 independent experiments were shown. Scale bar: 10 µm. LY dye transfer, initiated by scratching cells and thereafter stained with LY dye, was suppressed in linearly aligned ChAT KO HL-1 cells, as compared with control cells (vs. control: 28.0±4.0%; *P*<0.01, n = 10) (B−c). The non-neuronal cholinergic system in cardiomyocytes played a role in maintaining cell−cell communication via gap junction. Scale bar: 10 µm. (C) ChAT KO HEK cells with a few ACh (vs. control: 9.3±3.5%; *P*<0.01, n = 9; C−a) reciprocally increased MTT activity (vs. control: 384.8±20.8%; *P*<0.01, n = 9; C−b) and consumed more oxygen (vs. control: −239.4±26.4%; *P*<0.01, n = 9; C−c). (D) ChAT KO HEK cells also showed reciprocally lower ATP levels than control cells (control vs. ChAT KO: 765.1±56.9 vs. 425.7±18.7 mM/g protein; *P*<0.01, n = 6). (E) After inducing chemical hypoxia with 2 mM of CoCl_2_ more ChAT KO HEK cells died compared with control cells (vs. control: 26.6±2.33%; *P*<0.01, n = 7). (F) Similarly, ChAT KO HL-1 cardiac cells also consumed more ATP than control cells (control vs. ChAT KO: 271.9±28.2 vs. 83.1±8.8 mM/g protein; *P*<0.01, n = 7), indicating that the non-neuronal cardiac cholinergic system inhibited energy wasting. (G) Similar to the ChAT gene knockdown, the ChAT inhibitor HC-3 (10 µM) also increased MTT activities (G−a) and ATP consumption in rat myocardium-derived H9c2 cells (*P*<0.05, n = 4; G−b).

Permanent ChAT KO cells proliferated comparably with control cells, in which HEK293 cells were stably transfected with a LacZ gene knockout vector. However, re-inoculation of ChAT KO cells, after their agitation-induced detachment revealed a characteristic of these cells– they exhibited inefficient cell−cell communication compared with control cells (Figure 2A−a). After agitation, ChAT KO cells were easily dispersed as single cells, whereas control cells maintained their cell−cell interactions, and formed aggregates (Figure 2A−a). Weakened immunoreactive β-catenin signals were detected between ChAT KO cells, in great contrast to the more intense, stronger signals between control cells (Figure 2A−b). The lengths of β-catenin signals were decrease in ChAT KO cells (80.7±4.7% vs. control, *P*<0.05), and moreover, the rate of cells expressing intense β-catenin signals, which showed linear appearance, was decreased in ChAT KO cells (20.1±2.2%, *P*<0.05) compared with that in control cells (38.6±3.2%) (Figure 2A−b). In control cells, ACh-positive signals were detected on the cytoplasmic membrane; however, these signals were attenuated in ChAT KO cells (Figure 2A−c).

In addition to experiments using non-cardiac cells, ChAT KO HL-1 cells were also prepared; along with LacZ gene knockout cells (control cells) (Figure 2B). ChAT KO HL-1 cells had reduced Cx43 protein expression, as confirmed by western blot (ChAT: 33.0±5.2% vs. control, *P*<0.01; Cx43: 48.7±5.0% vs. control, *P*<0.01) and immunocytochemical analyses (Figure 2B−a). Cx43 positive signals were clearly detected between control cells, but were remarkably reduced in ChAT KO HL-1 cells (signal intensity: 41.6±5.1% vs. control, *P*<0.05; signal length: 25.8±1.5% vs. control, *P*<0.05) (Figure 2B−b), which suggested that cell−cell communication was disturbed. A dye transfer assay also revealed that LY dye was not efficiently transferred between ChAT KO HL-1 cells (Figure 2B−c). In contrast, the dye applied to control cells was remarkably transferred over a long distance (ChAT KO vs. control: 28.0±4.0% vs. 100.0±11.1%; *P*<0.01, n = 10), which suggested that the non-neuronal cholinergic system regulated a gap-junction function.

Taken together, these data suggest that the non-neuronal cholinergic system plays a role in cell−cell communication.

### 2. The Non-neuronal Cholinergic System Plays a Role in Preserving Energy Metabolism

To investigate additional roles of this system, we used ChAT KO HEK cells that had <10% of the ACh production level of control cells (control vs. ChAT KO: 100.0±8.7% vs. 9.3±3.5%; *P*<0.01, n = 9) in terms of their cellular energy metabolism (Figure 2C−a).

Even at comparable cell numbers, the MTT activity of ChAT KO HEK cells were more increased compared with that of control cells (384.8±20.8% vs. control; *P*<0.01, n = 9; 2C−b). Consistent with this, ChAT KO HEK cells consumed more oxygen than control cells (−239.4±26.4% vs. control; *P*<0.01, n = 9; Figure 2C−c). As a result, the ATP levels in ChAT KO HEK cells were significantly decreased (control vs. ChAT KO: 765.1±56.9 vs. 425.7±18.7 mM/g protein; *P*<0.01, n = 6; Figure 2D).

These results showed reciprocal changes between MTT activity and oxygen consumption and ATP levels and suggested that the non-neuronal cholinergic system negatively regulated cellular energy metabolism. In addition, this system maintained cellular ATP levels. Consequently, when oxygen utilization was interfered with using the chemical hypoxic mimetic cobalt chloride (2 mM), ChAT KO HEK cells were more susceptible to cell death compared with control cells, which remained intact even at this dose (control vs. ChAT KO: 100.0±0.4% vs. 26.6±2.3%; *P*<0.01, n = 7; Figure 2E).

Similarly, in HL-1 cells, the non-neuronal cholinergic system played an important role in regulating myocardiac energy metabolism. The ATP levels in ChAT KO HL-1 cells were significantly reduced compared with control cells (control vs. ChAT KO: 271.9±28.2 vs. 83.1±8.8 mM/g protein; *P*<0.01, n = 7; Figure 2F). These phenomena were also observed by other myocardium derived cells H9c2 cells, treated with a ChAT inhibitor HC-3 (10 µM), instead of ChAT gene knockdown, to inhibit ChAT activity without any morphological changes. HC-3 increased the MTT activity (Figure 2G−a) and reciprocally decreased the ATP level (control vs. HC-3: 92.0±1.0 vs. 61.0±1.1 mM/g protein; *P*<0.05, n = 4; Figure 2G−b), similar to the results with ChAT KO HL-1. These data even from different cell lines and modalities to suppress ChAT activity also suggest that the non-neuronal cholinergic system fundamentally suppresses oxygen consumption to preserve cellular ATP in cardiac cells.

### 3. The Non-neuronal Cholinergic System Protects Cells from Norepinephrine Toxicity

**Figure 3:**
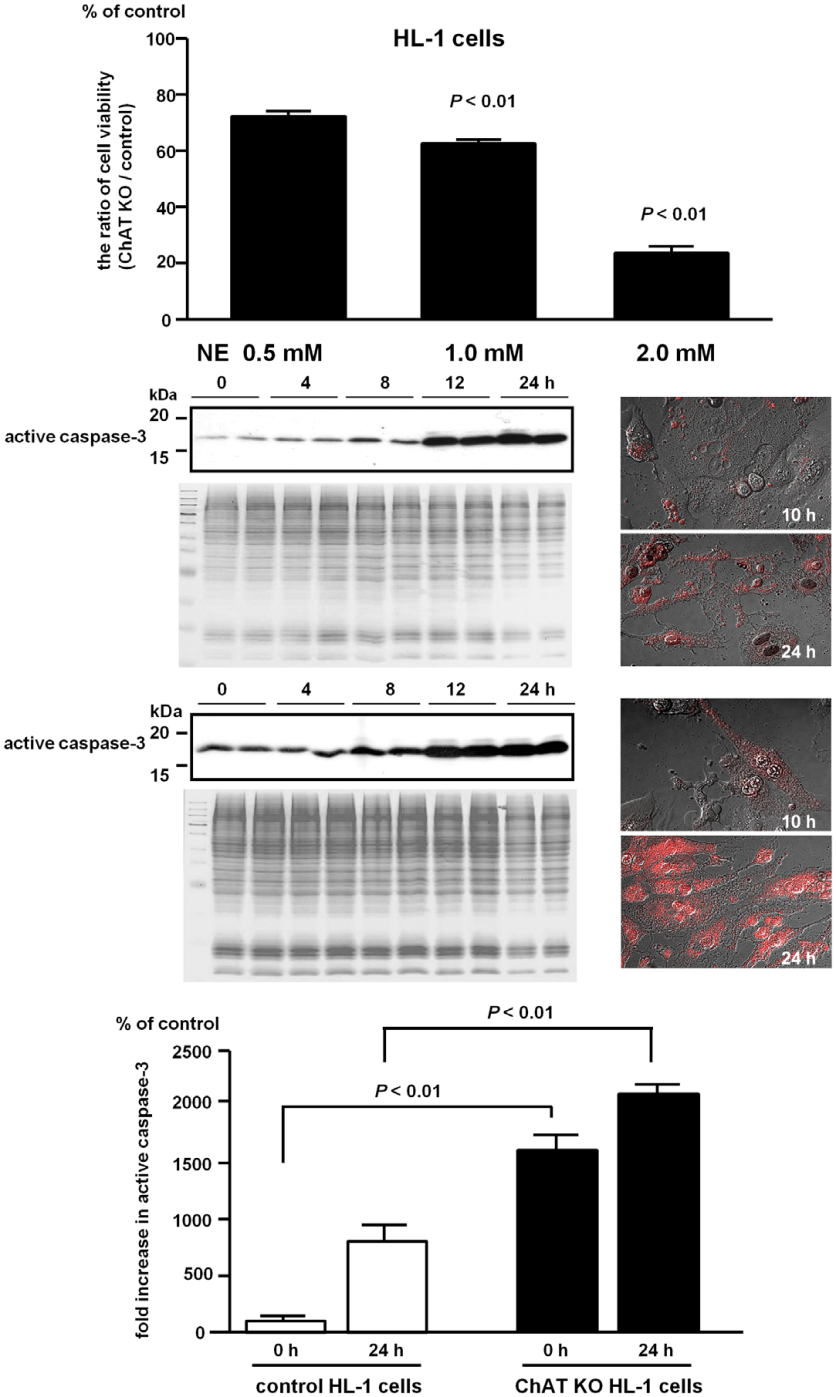
ChAT KO HL-1 cells are susceptible to norepinephrine (NE)-induced cell death. Cell viability of ChAT KO cardiac cells, as assessed by comparisons with control cells, was dose-dependently decreased by NE treatment, but control cells treated with 2 mM NE appeared to remain intact appearance (vs. control: 29.4±2.4%; *P*<0.01, n = 8). After treatment with 2 mM NE, ChAT KO cells produced more reactive oxygen species, as indicated by increased APF red fluorescence compared with control cells. Scale bar: 10 µm. ChAT KO cells increased active caspase-3 expression at basal condition (vs. control at 0 h: *P*<0.01, n = 6), and after NE treatment caspase-3 activation was further enhanced in ChAT KO cells (vs. control at 24 h: 2071±20.8%: *P*<0.01, n = 6), indicating that the system plays a role in protecting cardiomyocytes from NE toxicity.

ChAT KO HL-1 cells were treated with norepinephrine (NE) (Figure 3). In this experiment, we used the proportion of viable NE-treated ChAT KO HL-1 cells compared with control cells as an indicator of cell viability. Thus, 100% indicated that both cells were equally intact even in the presence of NE. As clearly demonstrated in Figure 3, NE dose-dependently decreased the cell viability of ChAT KO HL-1 cells (vs. control: 73.6±1.4% at 0.5 mM of NE, *P*<0.01, n = 8; 62.0±1.0% at 1.0 mM of NE, *P*<0.01, n = 8). NE at 2 mM resulted in increased death of ChAT KO HL-1 cells (vs. control: 29.4±2.4%; *P*<0.01, n = 8), which was accompanied by enhanced caspase-3 activity compared with control cells (vs. control: 2071±20.8% at 24 h; *P*<0.01, n = 6). In addition, ChAT KO HL-1 cells produced more reactive oxygen species during the cell death process induced by NE, as assessed by the ROS indicator APF. This increased production of ROS may have partly contributed to cell death. These results clearly indicate that the non-neuronal cholinergic system in cardiomyocytes is essential for protecting these cells from NE toxicity.

Taken together, all these *in vitro* data suggest that the non-neuronal cardiac cholinergic system contributes to maintaining cell−cell communication, negatively regulating cellular energy metabolism, and protecting cells from cell death.

### 4. In vivo Acceleration of the Non-neuronal Cholinergic System in Skeletal Muscle

**Figure 4:**
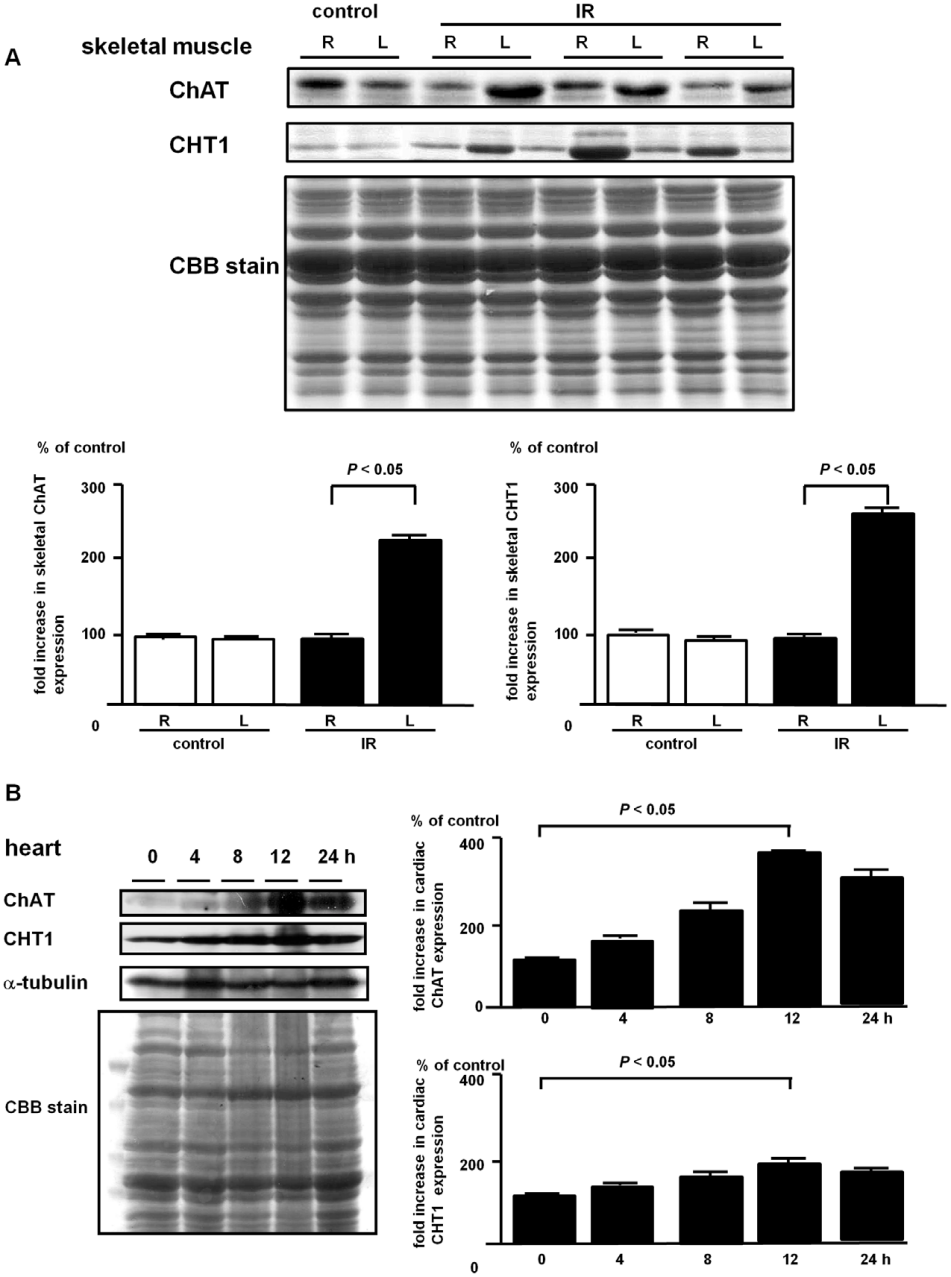
Hindlimb IR enhances ChAT and CHT1 expression in the affected hindlimb and in the heart. (A) Compared with the right hindlimb (R), the left hindlimb subjected to IR had increased protein expression of ChAT and CHT1 in tissue samples isolated from quadriceps femoris muscle within 16 h (*P*<0.05, n = 6, respectively). (B) The heart excised after IR also showed increased expression of both ChAT and CHT1, and the levels were peaked at 12 h (0 h vs. 12 h: *P*<0.05, n = 6, respectively) in the heart.

To investigate the regulatory mechanism of this system *in vivo*, we performed murine transient hindlimb ischemia-reperfusion (IR) by manually attenuating the left femoral artery flow in an intermittent fashion; 3 cycles of ischemia (5 min) with reperfusion (3 min), a protocol similar to preconditioning (Figure 4A). Sixteen hours after IR, the affected left quadriceps femoris muscle had remarkably increased protein expression of ChAT and CHT1 compared with the muscle from contralateral limb (vs. right; 211.5±1.1% in ChAT, *P*<0.05, n = 6; 288.7±1.4% in CHT1, *P*<0.05, n = 6). As already reported by our previous study, a murine *in vivo* reporter assay using a ChAT promoter vector, directly transfected to hindlimb skeletal muscles, not nerves, demonstrated that a non-neuronal cholinergic system was transcriptionally upregulated through an increased ChAT reporter activity in the skeletal muscles [11], suggesting that upregulated ChAT and CHT1 were more likely derived from skeletal muscle. Taken together with those previous results, this suggested that IR itself was a trigger to activate the skeletal (local) non-neuronal cholinergic system.

Of note, during this procedure, this system was also activated in the heart (Figure 4B), which suggested that IR could remotely activate the non-neuronal cardiac cholinergic system; however, IR itself did not affect murine heart rate to decrease 16 h after IR (data not shown). Cardiac ChAT protein expression was gradually increased within 24 h. The time−course of CHT1 protein expression in the heart followed a similar pattern as that of ChAT (0 h vs. 12 h: *P*<0.05, n = 6, respectively; Figure 4B). These data suggested that IR can upregulate the non-neuronal cholinergic system not only in an affected (local) organ, but also in a remote organ, e.g., the heart, however, not associated with the heart rate-decreasing effect.

### 5. Hindlimb Ischemia-reperfusion in vivo Salvages the Whole Heart from Global Ischemia through the Cardiac Non-neuronal Cholinergic System

**Figure 5:**
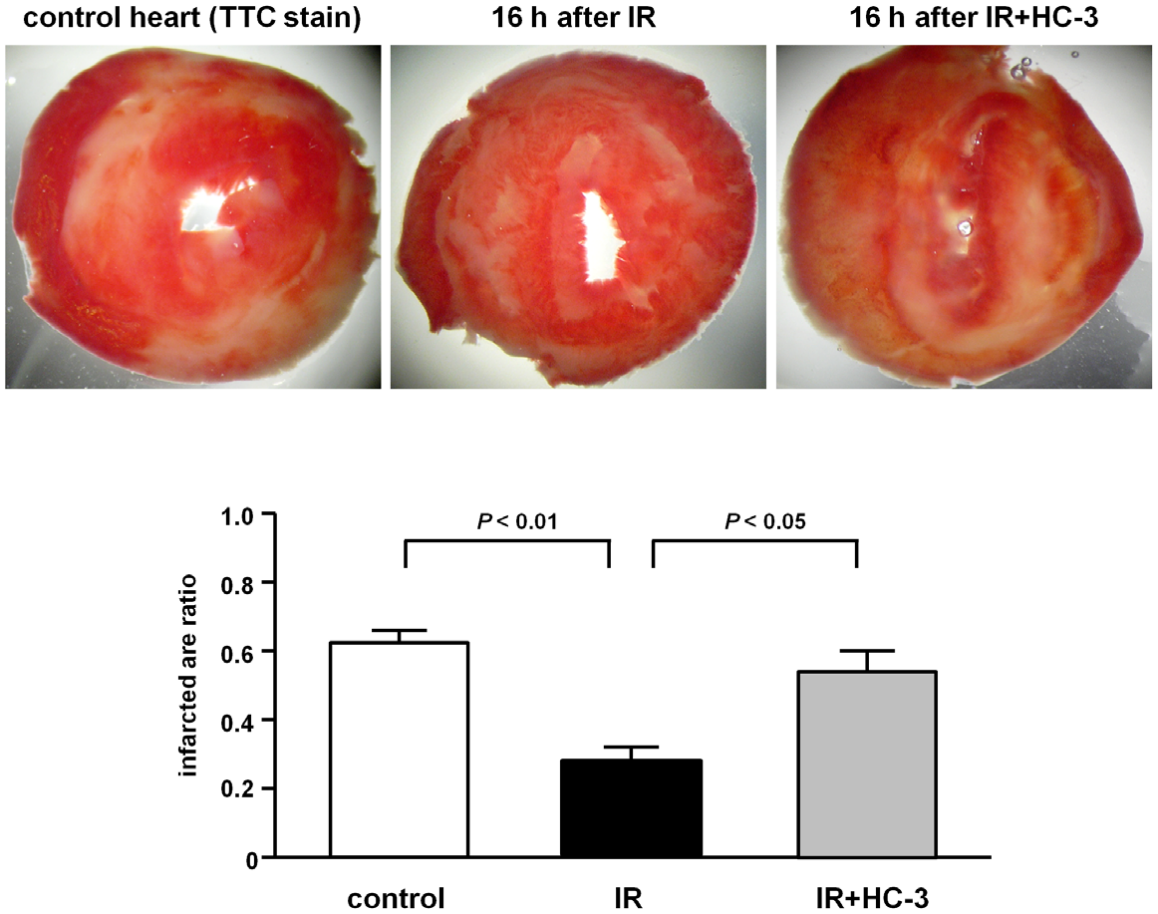
IR salvages the heart from global ischemia-induced myocardial infarction through ChAT activity. Compared with controls without IR (0.61±0.03, n = 7), IR protected the heart from global ischemia and decreased the infarcted area (0.26±0.03; *P*<0.01, n = 7) as shown by TTC staining. Pretreatment with HC-3 before IR completely abolished the cardioprotective effects by IR (0.56±0.03 vs. IR; *P*<0.05, n = 7). Scale bar: 100 µm.

To confirm that IR protected the heart from global ischemia, a mouse heart was excised 16 h after IR, perfused using a Langendorff apparatus, and subjected to global IR, including 30 min ischemia and 60 min reperfusion (Figure 5). A Langendorff apparatus connected to the excised heart without vagal nerve could exclude the possibility of neuronal ACh secretion. As a consequence of reperfusion injury, the ratio of the infarcted area to the entire area in control mice, that did not receive IR, was 0.61±0.03 (n = 7). In contrast, the ratio of the infarcted area in the hearts excised from mice with IR 16 h later was significantly reduced to 0.26±0.03 (vs. control; *P*<0.01, n = 7), which confirmed a remote protective effect of IR on the mouse heart.

This phenomenon is well known as a late phase of preconditioning. However, pretreating IR-treated mice with the ChAT inhibitor HC-3 before IR removed this myocardial infarction sparing effect, as the ratio of the infarcted area returned to a level comparable with that of the control mice at 0.56±0.03 (vs. IR; *P*<0.05, n = 7). However, HC-3 administered before IR did not increase murine heart rate after 16 h, before the heart was excised. These results suggested that the remote cardiac protection effect was mediated by upregulating ChAT in the heart because HC-3 canceled this effect.

### 6. Mechanisms Underlying Cardioprotection by the Remote Effect

**Figure 6:**
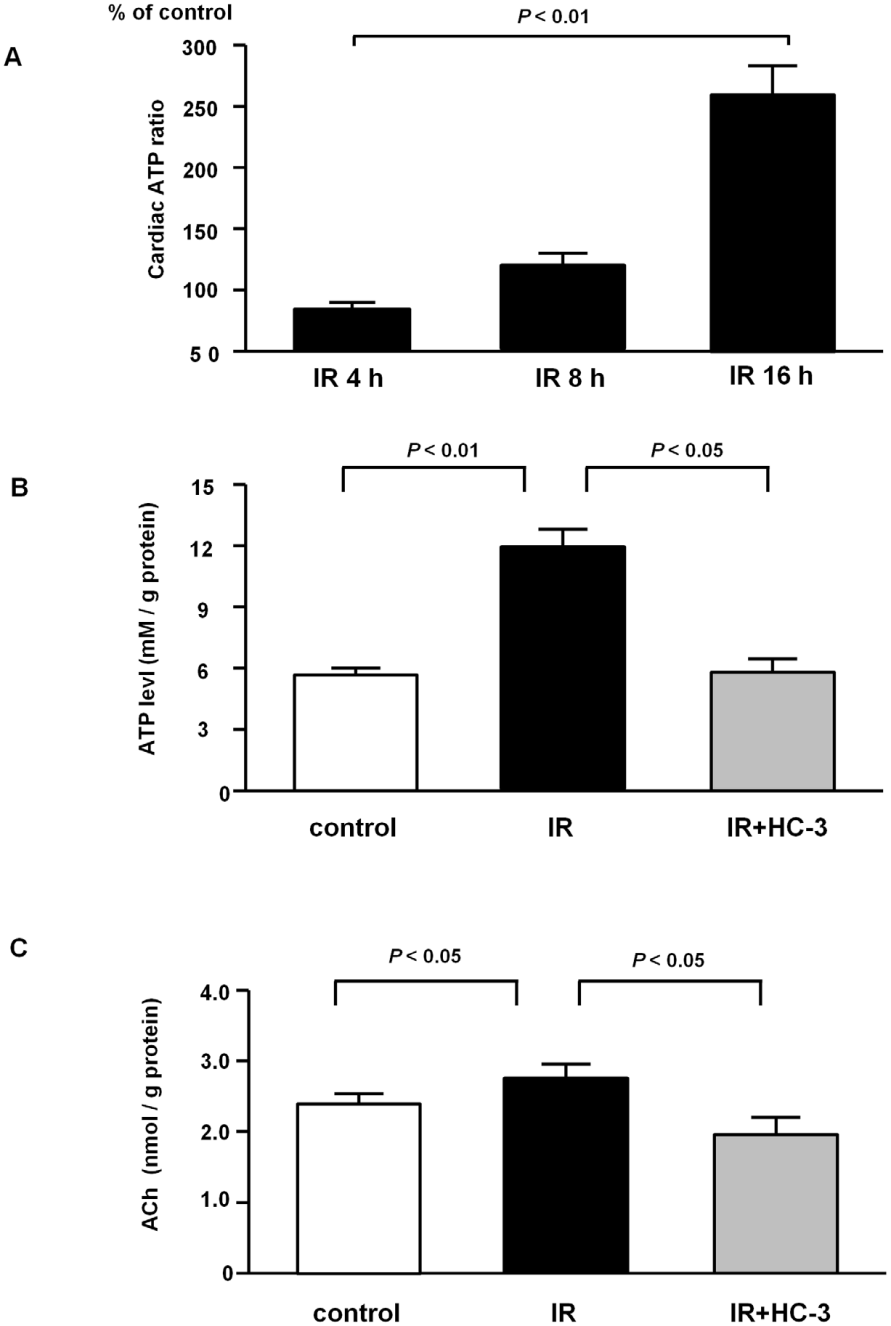
The remote effect of IR of the hindlimb contributes to activating the non-neuronal cholinergic system in the heart, which sustains cardiac ATP levels. (A) IR gradually increased the ATP level in the heart within 16 h (256.4±13.1% vs. IR 4 h; *P*<0.01, n = 8). (B) The increased cardiac ATP level induced by IR (IR: 11.5±0.9 mM/g protein vs. control: 5.9±0.5 mM/g protein; *P*<0.01, n = 7) was suppressed by pretreatment with HC-3 before IR (HC-3: 6.4±1.0 mM/g protein vs. IR; *P*<0.05, n = 7). (C) Under these conditions, the cardiac ACh level also followed these trends, as the ACh level was increased by IR (IR: 2.78±0.17 nmol/g protein vs. control: 2.32±0.12 n mol/g protein; *P*<0.05, n = 7) and attenuated by HC-3 (HC-3: 2.14±0.07 vs. IR; *P*<0.05, n = 7) in parallel with the ATP level.

Hearts were isolated at 4 h, 8 h and 16 h after IR and heart ATP levels were determined. At 16 h, the heart ATP levels were significantly increased to peak (256.4±13.1% vs. IR at 4 h, *P*<0.01, n = 8), which suggested that IR definitely caused the ATP level to increase in the heart (Figure 6A). Therefore, in the following experiments, the heart was sampled 16 h after IR.

To investigate whether an increased heart ATP level could be attributed to upregulating the cardiac non-neuronal cholinergic system, HC-3 was administered before IR. As shown in Figures 4 and 6C, IR resulted in upregulated ChAT protein expression in the heart and increased the cardiac levels of both ACh (IR: 2.78±0.17 nmol/g protein vs. control: 2.32±0.12 n mol/g protein; *P*<0.05, n = 7; Figure 6C) and ATP (IR: 11.5±0.9 mM/g protein vs. control: 5.9±0.5 mM/g protein; *P*<0.01, n = 7; Figure 6B). However, the ChAT inhibitor HC-3 surprisingly attenuated the increase in the cardiac ATP levels to control levels (HC-3: 6.4±1.0 mM/g protein vs. IR; *P*<0.05, n = 7; Figure 6B). This suggested that the cardiac non-neuronal cholinergic system was involved in maintaining the heart ATP level. ACh level measurement in the hearts from mice subjected to IR also supported this issue. Pretreatment with HC-3 completely suppressed the increased cardiac ACh levels (HC-3: 2.14±0.07 vs. IR; *P*<0.05, n = 7; Figure 6C). Based on the results, thus far presented, the non-neuronal cardiac cholinergic system played a critical role in conserving ATP levels, probably through delayed or suppressed ATP consumption, because this system was suggested to be involved in attenuating oxygen consumption by our previous study [11].

## Discussion

In our recent study, we reported for the first time that cardiomyocytes themselves synthesized ACh and that cardiomyocytes were equipped with a non-neuronal cardiac cholinergic system [11]. However, additional studies are necessary to determine whether this system has beneficial effects on cardiomyocytes. It has already been shown that several cell types, including lymphocytes and endothelial and epithelial cells, except for cardiomyocytes, have their own non-neuronal cholinergic systems with their specifically local functions [13], [16]–[18]. However, no study has clarified the biological functions of these systems, particularly studies that focused on a cardiomyocyte-derived non-neuronal cholinergic system involving its effects on anti-apoptosis and anti-hypoxia.

As shown in the present study, investigating a beneficial function of this system in cardiomyocytes suggested a novel therapeutic modality for cardiovascular diseases, because the system in cardiomyocytes is indispensable for maintaining a gap junction function and also responsible for regulating the cellular ATP levels. Based on the previous [1], [10], [11] and present studies, ACh from cardiac or/and neuronal sources upregulated post-translationally the HIF-1α protein level in cardiomyocytes during a normoxic condition, downregulated mitochondrial function of cardiomyocytes to suppress oxygen consumption. Therefore, the non-neuronal cardiac cholinergic system seemed to decrease ATP consumption. Furthermore, our unpublished data that ChAT overexpressing cells increased the HIF-1α protein level strongly supported this interpretation.

The therapeutic targets of this system may be unique in terms of modulation of cellular energy metabolism and distinct from the targets of conventional therapies, including anti-neurohumoral factor therapy. Vagal nerve stimulation has been shown to be another promising novel modality against cardiovascular diseases [2], [4], [19]–[21]. However, how vagal nerve stimulation, even with its poor innervation of cardiac ventricles, can prevent heart remodeling during chronic heart failure is yet to be determined [22], [23].

We have been intensively investigating the pleiotropic effects of ACh, including heart rate-independent anti-hypoxic and anti-apoptotic effects through a PI3K/Akt/HIF-1α induction pathway [1], [4], [6], [10] and anti-arrhythmogenic effects [2], [3]. ACh is released from the vagal nerve end, which predominantly innervates the atrial conduction system but not the ventricles, and is rapidly degraded by acetylcholinesterase. Therefore, it would be difficult to expect that nerve end-derived ACh alone could be delivered far from these nerve terminals into the whole heart. Thus, a non-neuronal cardiac cholinergic system identified in our recent study could provide the missing link between poor vagal nerve innervation in cardiac ventricles and the promising effects of vagal nerve stimulation in chronic heart failure [11]. In that previous study, we confirmed that the system negatively regulated mitochondrial function and suggested its fundamental biological significance. In addition, we found that this system was activated by a positive feedback through a muscarinic receptor, leading to ACh or a muscarinic receptor agonist-induced ACh synthesis in cardiomyocytes. We further demonstrated that a specific acetylcholinesterase inhibitor, donepezil, possessed a unique function of activating this cardiac system, unlike another acetylcholinesterase inhibitor, physostigmine [11].

Although a non-neuronal cardiac cholinergic system provided apparent positive outcomes for cells in the current *in vitro* study through regulating an energy metabolism status, the following issues remained to be resolved: 1) whether it would attenuate the progression of pathological insults; and 2) how it was activated by means other than drugs. Therefore, we further investigated these issues in the present study.

Using a gene knockout system, ChAT KO non-cardiac cells were generated to eliminate ACh synthesis. These cells were useful in assessing a ChAT-specific phenotype due to the high transfection efficiency and high protein expression potency and were compared with negative control cells silenced with LacZ-specific siRNA. It was indicated first that, the non-neuronal cholinergic system was involved in cell−cell communication because less β-catenin immunoreactivity was observed in the plasma membranes and simple agitation of these cells resulted in the loss of cell−cell interactions and increased cell scattering. β-catenin is known to be localized in the plasma membrane and form a complex with other molecules responsible for cell−cell communication, including cadherin and connexin [24]–[26]. Therefore, ChAT KO attenuated a β-catenin signal, which was responsible for anchoring membranes between cells, suggesting that ACh played a role in maintaining cell−cell interactions. Moreover, the same phenotypes of poor cell−cell communication with decreased protein expression of connexin among CHAT KO cardiac cells also supported a critical role for ACh derived from the system. These comparable phenotypes shared between non-cardiac and cardiac cells suggest that the non-neuronal cardiac cholinergic system plays a fundamental biological role in cardiomyocytes.

Second, ChAT KO cardiac cells exhibited a phenotype critical for regulating energy metabolism, as previously reported [11]. Both cardiac and non-cardiac ChAT KO cells had increased MTT activity, and as a result, the ATP levels in these cells were lower than those in control cells (Figure 2C, 2D and 2F). This suggested that ChAT plays an inhibitory role in energy metabolism. These results were comparable with those of ChAT inhibitor-treated H9c2 cells (Figure 2G). Based on these similar results from several cardiac cell lines with a different origin, taken together with the ACh/HIF-1α/mitochondria cascade identified by us [1], [10], the non-neuronal cardiac cholinergic system is suggested to constitutively suppress ATP consumption and subsequently maintain cellular ATP levels.

We also found that this energy metabolism suppression effect salvaged ChAT KO HEK cells from chemical hypoxic stress (Figure 2E) and was also involved in cardioprotection when cardiomyocytes were challenged by a surge of norepinephrine (NE). Without this non-neuronal cardiac cholinergic system, cardiac HL-1 cells readily underwent apoptosis due to NE and produced more ROS. In fact, loss of this system caused cells to become highly susceptible to NE toxicity. Taken together, the negative regulation of ATP consumption by this non-neuronal cardiac cholinergic system may contribute to protecting cells from ROS and apoptosis.

Third, our results indicated that this system was beneficial for cell survival *in vivo* as well as *in vitro*. In a murine transient hindlimb IR model, it was found that protein expression of cholinergic system components, ChAT and CHT1, were upregulated in the affected left hindlimb within 24 h compared with the contralateral limb. Possible contamination of neurons might have interfered with the understanding that those ChAT and CHT1 proteins were derived from the skeletal muscle alone and it was practically not possible to differentiate from which they were derived between the skeletal muscle and neurons. However, the extremely larger muscular sample volume than neuronal one and the upregulation of the muscular non-neuronal cholinergic system evaluated by an *in vivo* reporter assay [11] prompted us to understand that those were predominantly derived from the skeletal muscle. These results suggested that *in vivo* IR activated a local, non-neuronal cholinergic system in the left quadriceps femoris muscle through *de novo* synthesis of these components. Surprisingly, IR also induced the activation of a non-neuronal cholinergic system in a remote organ, the heart, as shown in Figure 4. After this procedure, the protein expression patterns of cardiac ChAT and CHT1 were similar to those in the left ischemic hindlimb. Due to the poor cholinergic innervation in the murine heart [22], [23] and based on the evidence that cardiomyocytes synthesized a comparable amount of ACh [11], cardiac ChAT and CHT1 were also considered to be predominantly from cardiomyocytes.

These results provided a glimpse of a late phase, remote, preconditioning effect induced by IR [27]–[30]. However, this was not the point that we addressed in this study. Rather, we deduced a novel concept from the current study in that activation of a non-neuronal cardiac cholinergic system was enhanced by the IR procedure. As was clearly shown, IR prevented the Langendorff-perfused heart from global ischemia; however, this effect was completely abolished by pre-treatment with HC-3, a ChAT inhibitor. As shown in Figure 5, global ischemia-induced myocardial necrosis, which was remarkably attenuated by this procedure, was reversed by HC-3. These results definitely support our speculation.

IR induction of cardiac ChAT and CHT1 expression was accompanied by increased cardiac ACh levels, which gradually increased within 24 h after IR, however, heart rate of mice from both groups of IR and IR with HC-3 was not affected during the experiments. These findings suggest that IR-induced activation of the system was mediated by the *de novo* synthesis of each components of the system. This activated non-neuronal cardiac cholinergic system increased the heart ATP levels more than that in the HC-3 pretreated heart.

The precise triggers for the activation of the system through IR have not yet been fully studied at present, and various speculations regarding them are raised including humoral or neurogenic factors. However, the system activated by IR resulting in increased cardiac ACh synthesis was involved in cardioprotection. Increased synthesis of ACh in the heart could activate the survival signal through the PI3K/Akt/HIF-1α pathway, which efficiently upregulates oxygen-independent energy metabolism, i.e., anaerobic or glucose metabolism; in contrast, ChAT knockdown further increased oxygen consumption, both of which also supported our speculation. In conjunction with our other results discussed above, it is suggested that activation of a non-neuronal cardiac cholinergic system suppresses energy metabolism by increasing ACh levels in the heart and consequently conserving adequate ATP levels in the heart. Thus, sustained cardiac ATP during ischemia, probably due to the delayed consumption, could prevent the myocardium from global infarction. The tight linkage between IR, increased cardiac ACh, sustaining cardiac ATP, and downregulated oxygen consumption could prevent the heart from the unproportinoal energy demand, which may cause cellular energy depletion. This system, therefore, plays an indispensable role in protecting cells from energy depletion-induced cell death in the pathological situations.

We have previously investigated the pleiotropic effects of ACh with regard to the cardiovascular system and reported that (1) ACh maintains the total protein and phosphorylation levels of connexin 43 in the heart, which is rapidly degraded through the ubiquitin−proteasome system by cardiac ischemia or hypoxia, and inhibits ventricular arrhythmia induced by myocardial infarction [2], [3]; (2) ACh upregulates cardiac ischemic tolerance through non-hypoxic induction of HIF-1 by survival signals and suppression of mitochondrial function [1], [10]; (3) ACh accelerates angiogenesis *in vitro* and *in vivo* [7]; (4) ACh positively regulates ACh synthesis through muscarinic receptors in cardiomyocytes [11]; and (5) an HIF-1α-regulated gene represses the transcription of mitochondrial transcription factor A and results in suppression of cellular oxygen consumption [10]. These pleiotropic actions of ACh strongly support our current conclusion that a non-neuronal cardiac cholinergic system modulates energy metabolism partially by sparing mitochondria from consuming more oxygen and alternatively by preventing cells from wasting ATP.

Recently, a remote effect of preconditioning was addressed in terms of protecting an organ from ischemia. Several clinical trials demonstrated that remote preconditioning, a simple, non-invasive, and cost-free intervention, could improve clinical outcomes in cardiovascular diseases [27]–[30]. Although several mechanisms have been hypothesized, the key molecules underlying these effects are yet to be determined [30]. Therefore, the current study provides another underlying mechanism, which may integrate previous key players through activation of a non-neuronal cardiac cholinergic system.

In conclusion, a non-neuronal cholinergic system plays a pivotal role in efficiently regulating cardiac energy metabolism *in vivo,* and constitutively protects the heart from cell death resulting from catecholamine exposure. Moreover, it is possible that the induction of this system results in enhanced endogenous cardioprotective potency through ChAT.
